# Electronic Cigarette Use and Smoking Abstinence in Japan: A Cross-Sectional Study of Quitting Methods

**DOI:** 10.3390/ijerph14020202

**Published:** 2017-02-17

**Authors:** Tomoyasu Hirano, Takahiro Tabuchi, Rika Nakahara, Naoki Kunugita, Yumiko Mochizuki-Kobayashi

**Affiliations:** 1 Center for Cancer Control and Information Services/Center for Public Health Sciences, National Cancer Center, Tokyo 104-0045, Japan; ymochizu@ncc.go.jp; 2 Center for Cancer Control and Statistics, Osaka Medical Center for Cancer and Cardiovascular Diseases, Osaka 537-8511, Japan; tabuchitak@gmail.com; 3 National Cancer Center Hospital, National Cancer Center, Tokyo 104-0045, Japan; rinakaha@ncc.go.jp; 4 Department of Environmental Health, National Institute of Public Health, Saitama 351-0197, Japan; kunugita@niph.go.jp

**Keywords:** electronic cigarettes, smoking cessation, smoking cessation therapy, quitting methods

## Abstract

The benefit of electronic cigarettes (e-cigarettes) in smoking cessation remains controversial. Recently, e-cigarettes have been gaining popularity in Japan, without evidence of efficacy on quitting cigarettes. We conducted an online survey to collect information on tobacco use, difficulties in smoking cessation, socio-demographic factors, and health-related factors in Japan. Among the total participants (*n* = 9055), 798 eligible persons aged 20–69 years who smoked within the previous five years were analyzed to assess the relationship between the outcome of smoking cessation and quitting methods used, including e-cigarettes, smoking cessation therapy, and unassisted. E-cigarette use was negatively associated with smoking cessation (odds ratio (OR) = 0.632; 95% confidence interval (CI) = 0.414–0.964) after adjusting for gender, age, health-related factors, and other quitting methods. Conversely, smoking cessation therapy (i.e., varenicline) was significantly associated with smoking cessation (OR = 1.885; 95% CI = 1.018–3.492) in the same model. For effective smoking cessation, e-cigarette use appears to have low efficacy among smokers in Japan. Allowing for the fact that this study is limited by its cross-sectional design, follow-up studies are needed to assess the prospective association between e-cigarette use and smoking cessation.

## 1. Introduction

Electronic cigarettes (e-cigarettes) are devices that do not burn tobacco leaves, but instead vaporize liquid for inhalation by the user [1,2]. E-cigarettes have recently been introduced into the Japanese market as an alternative to smoking, irrespective of whether nicotine is included or not [3]. 

Although e-cigarettes have been shown to be attractive to smokers who want to quit, there is conflicting evidence as to whether e-cigarettes are effective for smoking cessation. Numerous advantages and disadvantages to their use have been identified for both smoking cessation and reduction [4,5,6,7]. Advocates for e-cigarettes insist that the product has potential, and that if their development were to continue, e-cigarettes would allow smokers to switch to a safer product (i.e., harm reduction), and thereby eventually end cigarette use completely [4,5,6,7,8]. In contrast, critics in favor of restrictions on e-cigarettes argue that they carry the risk of increasing cigarette use by re-normalizing smoking (i.e., reducing the motivation of smokers to quit completely) [9,10,11].

Evaluating the effect of e-cigarettes in smoking cessation is essential for public health policy. Two meta-analyses results from multiple clinical trials to assess whether e-cigarette use is associated with smoking cessation [6,7]. Although these studies showed that e-cigarette use improved long-term smoking cessation, they did not compare e-cigarette users to a control group that did not use e-cigarettes. On the contrary, two meta-analyses have found that e-cigarette use is not associated with significant depression during smoking cessation [10,11].

Change in prevalence of e-cigarette use in England has been positively associated with quit attempt success rates and negatively associated with prescription nicotine replacement therapy (NRT) use [12]. Furthermore, in a cross-sectional population survey among smokers in England trying to quit without professional support, self-reported e-cigarette users were found to be more likely to abstain from smoking than those who used either an over-the-counter licensed nicotine replacement therapy (NRT) product or no aid at all [13]. However, this study did not include smokers who did not try to quit smoking, making it incomparable to most e-cigarette studies. Moreover, the cross-sectional nature of this study allows for limited capacity for causal influence. In the United States, e-cigarette use and smoking cessation were evaluated, but there was no evidence of a direct positive association between e-cigarettes and cessation [14,15]. Therefore, most studies of the effect of e-cigarette use on smoking cessation have reported varying results.

In Japan, there has yet to be a nationwide study on e-cigarette use and smoking cessation [16]. Here we examined the association between e-cigarette use and smoking cessation compared with NRT, smoking cessation therapy, and no aid in a Japanese population.

## 2. Materials and Methods 

### 2.1. Participants

A cross-sectional online survey was conducted between 31 January and 17 February 2015. Participants (*n* = 106,200) were randomly sampled from a nationwide online panel (*n* = 2,278,733) maintained and provided by Rakuten Research, a commercial research provider. Panel members were skewed but covered all social categories (such as education, housing tenure, and marital status) defined by the Census of Japan, and provided written comprehensive agreement to participate in various investigative projects [3]. The first 9000 respondents (actually 9055, including a concurrent excess of 55) were recruited, consisting of 500 people aged 15–19 years and 800 people aged 20–29, 30–39, 40–49, 50–59, and 60–69 years of each sex, such that an age–gender balanced sample was obtained. In this survey, panel members consented after reading about the procedures of the study: that they would be asked about their present use of combustible tobacco and other emerging tobacco products, the age at which they first attempted to quit smoking, as well as demographic, socioeconomic, and health-related information. At the time of registration, respondents were required to provide information such as age, gender, occupation, and residence, and to agree to participate in the study. The survey was closed when the target number of respondents for each age–gender category was reached. Participation rate of respondents was 8.5% (9055/106,200). This procedure was considered the equivalent of written informed consent and was approved by the Research Ethics Committee of Osaka Medical Center for Cancer and Cardiovascular Diseases (1412175183). 

Among the total respondents (*n* = 9055), we selected smokers with a smoking history in the previous 5 years or more to analyze potential determinants of smoking cessation within the previous 5 years. Former regular smokers were asked, “How old were you when you quit smoking?” and this was used to calculate the period of smoking cessation by subtracting the quit age from the current age; if this was longer than 5 years, the respondent was excluded from the analysis. For current regular smokers, the question, “How old were you when you started smoking?” was used to assess the duration of smoking, by subtracting the start age from the current age. When the smoking period was 4 years or more, the respondent was deemed to fall under the category of those “who smoked as of 5 years ago” and was included in the analysis. Of the 3117 smokers (1222 current smokers and 1895 former smokers), 1512 people (1169 current smokers and 343 former smokers) smoked 5 years prior to the survey, 873 had quit smoking more than 5 years prior to the survey, 75 had started smoking within the previous 4 years, and 657 had not smoked cigarettes. Given the very low percentage (0.5%) of the Japanese population who start smoking in their early teens, we excluded respondents (*n* = 6) who were aged less than 15 years in the previous 5 years (i.e., under 20 years old at the time of this survey) [17]. Furthermore, we also excluded smokers who responded that they “have never intended to quit smoking” (*n* = 636), because smokers with no intention of quitting were considered incomparable to those who did intend to quit. Respondents with discrepancies in their answers to the questionnaire (*n* = 73) were also excluded. After these exclusions, the analysis was conducted in 798 persons aged 20–69 years who smoked within the previous 5 years and had intended to quit smoking at least once. 

### 2.2. Present Smoking Status and Cessation

Panel members were also asked to “Please choose your current status for paper-wrapped and roll-your-own cigarettes separately.” The response options presented were “never used”, “former non-regular user”, “former regular user”, and “current user”. Respondents who currently smoked combustible tobacco (paper-wrapped and/or roll-your-own cigarettes) were considered current smokers. However, the prevalence of roll-your-own cigarettes was only 0.4% (35/9055). Since roll-your-own cigarettes are similar in form and usage to paper-wrapped cigarettes, and only 4 people were using roll-your-own cigarettes without having used paper-wrapped cigarettes, paper-wrapped and/or roll-your-own cigarettes were combined and defined under combustible tobacco. Those who reported former regular use and did not currently smoke either type of cigarette were considered former smokers. We compared responses from current and former smokers, since these were considered “unsuccessful quitters” and “successful quitters”, respectively, within the previous 5 years.

### 2.3. Quitting Methods Used to Date

Current and former smokers were asked if they had ever attempted any of the following seven methods to quit smoking by responding Yes or No to each item: (1) nicotine gum bought over the counter, (2) nicotine patches bought over the counter, (3) e-cigarettes, (4) smoking cessation therapy (behavioral therapy for patients in an outpatient setting) for nicotine dependence (without medication), (5) smoking cessation therapy (medication, not including nicotine; i.e., varenicline), (6) smoking cessation therapy (medication, including nicotine; e.g., nicotine patches), and (7) unassisted cessation methods (including utilization of books). 

In Japan, the products available are nicotine gum and patches; nicotine gum is bought over the counter only, and nicotine patches are available both over the counter and on prescription [18]. Varenicline is the only oral smoking-cessation drug that has been approved and listed in the National Health Insurance price list, and has been widely prescribed by clinics that provide smoking cessation therapy [18,19]. If more than one year has passed since the first therapy, it can be re-administered under insurance coverage, so it may be possible to employ multiple methods within a five-year period.

(1) and (2) were defined as “NRT bought over-the-counter” and (4) and (6) were categorized as “Others” in smoking cessation therapy for nicotine dependence. 

### 2.4. Other Variables

Information on gender, current age, marital status (married, single, and widowed/divorced), age at start of smoking (younger than 20 years/20 years or older), and medical history (cancer and/or cardiovascular diseases, hypertension, and diabetes mellitus) was obtained from questionnaire responses.

### 2.5. Statistical Analysis

Chi-squared tests were used to compare the differences between current and former smokers (present smoking status) over the previous five years according to each characteristic variable. We used logistic regression models to assess the association between quitting methods conducted to date and smoking cessation (present smoking status) over the previous five years. In Model 1, gender- and age-adjusted odds ratios (aORs) and 95% confidence intervals (CIs) for smoking cessation were calculated. Model 2 was a multivariable logistic regression model which adjusted for the potential confounding factors of gender, current age, age at smoking initiation, marriage, and medical history [20]. Sensitivity analysis was conducted by repeating these analyses using “recent 4-year smokers” and “recent 3-year smokers”. All statistical analyses were performed using SAS version 9.2 (SAS Institute Inc., Cary, NC, USA).

## 3. Results

Characteristics such as quitting methods conducted to date, gender, and age group according to present smoking status of combustible tobacco over the previous five years are shown in Table 1. At the time of the survey, 545 (68.3%) of 798 smokers continued to smoke while 253 (31.7%) had quit. The most commonly used quitting methods (not mutually exclusive) among current and former smokers (present smoking status) were unassisted (81.8% and 87.0%), NRTs (27.7% and 26.1%), and e-cigarettes (22.0% and 15.4%, respectively). 

Results of logistic regression models for smoking cessation for the previous five years are shown in Table 2. E-cigarette use was negatively associated with smoking cessation (OR = 0.629; 95% CI = 0.415–0.954; Model 1) compared to no e-cigarette use. In a multivariable logistic regression model, e-cigarette use was negatively associated with smoking cessation after adjustment for potential confounding factors such as socio-demographics, smoking, and medical history (OR = 0.632; 95% CI = 0.414–0.964; Model 2). Conversely, use of smoking cessation therapy (medicine not including nicotine; i.e., varenicline) was significantly associated with smoking cessation (OR = 1.885; 95% CI = 1.018–3.492; Model 2). Compared to married smokers, unmarried (OR = 0.610; 95% CI = 0.409–0.910) or divorced/widowed (OR = 0.434; 95% CI = 0.240–0.786) smokers were negatively associated with smoking cessation.

In sensitivity analyses (see Table S1), similar results were obtained for smokers over the previous four years. Statistical significance decreased in the sensitivity analysis using the previous three years of smoking; however, this result might be attributable to the small sample size in this study.

## 4. Discussion

This study is the first survey in Japan to report the association between e-cigarette use and smoking cessation of combustible cigarettes among smokers who tried to quit smoking, compared to other cessation methods. E-cigarette use was negatively associated with smoking cessation after adjustment for gender, current age, age of smoking initiation, marital status, medical history, and other cessation methods. Conversely, medical cessation therapy with varenicline, which is covered by the National Health Insurance program in Japan, was significantly associated with smoking cessation in the same model. 

A recent study indicated that the magnitude and significance of the estimated association between e-cigarette use and cessation were dependent upon the choice to include a model with and/or without the use of other cessation aids in the analytical approach [21]. In this previous study, although e-cigarette use was negatively associated with abstinence after adjustment for baseline characteristics (adjusted odds ratio (aOR) = 0.68; 95% CI = 0.53–0.87), the association was no longer significant after additional adjustment for use of other cessation aids at 3 months follow-up (aOR = 0.83; 95% CI = 0.63–1.10).

In contrast, a systematic review of 15 cohort studies, 3 cross-sectional studies, and 2 clinical trials reported that e-cigarette use was negatively associated with quitting cigarettes (OR = 0.72; 95% CI = 0.57–0.91), irrespective of whether or not e-cigarette users had an interest in quitting cigarettes [11]. Another review showed a similar negative association for quitting (pooled OR = 0.61; 95% CI = 0.50–0.75) between users and non-users of e-cigarettes [10]. Thus, our observation of a negative association between e-cigarette use and quitting cigarettes is consistent with these reviews, but differed in the magnitude and significance of e-cigarette use on smoking cessation. These differences may likely have resulted from our exclusion of participants who reported never having had the intention to quit smoking.

Treatment of tobacco use and dependence is mandated by the World Health Organization (WHO) Framework Convention on Tobacco Control (FCTC), Article 14, as a key component of a comprehensive tobacco control strategy [1]. Each party of the FCTC is required to take effective measures to promote the cessation of tobacco use and provide adequate treatment for tobacco dependence (known as “O” of the MPOWER measures). In Japan, the National Health Insurance program has covered smoking cessation therapy and smoking cessation drugs in out-patient settings since 2008 [18]. There is now considerable evidence that NRT and varenicline use are associated with increased odds of quitting compared with a placebo [22], with odds ratios of 1.84 (95% CI = 1.71–1.99) and 2.88 (95% CI = 2.41–3.47), respectively. Our finding that varenicline was associated with higher smoking cessation rates than NRT and other quitting methods is consistent with the findings of previous studies. In Japan, the usage rate of NRT for smoking cessation is 16.6% and that for smoking cessation therapy is 7.4% [19], which is congruent with the results of our survey where the most commonly used quitting methods (not mutually exclusive) among current and former smokers were unassisted (81.8% and 87.0%) and NRTs (27.7% and 26.1%, respectively).

Although smoking cessation interventions such as NRT and varenicline are effective, the majority of smokers who quit successfully do so unassisted, without the use of interventions [23]. Further, we found that smokers who attempted to quit with e-cigarettes were significantly less likely to succeed than those who chose over-the-counter NRT and/or tried to quit without medical assistance. While e-cigarettes might positively affect public health by simply encouraging quitting, they might negatively affect it if they distract smokers from more effective quitting methods. Our results therefore suggest that even if e-cigarettes contribute slightly to smoking cessation, they are less likely to make a major contribution to the overall reduction of smoking, and should not be recommended or promoted as a smoking cessation aid. Implementation of an appropriate policy for e-cigarettes in Japan will require further evaluation of which quitting methods, including e-cigarettes, are associated with successful smoking cessation. Without evidence of an effect on smoking abstinence, any recommendation to promote e-cigarettes in the same manner as other medically proven methods such as varenicline treatment is premature.

In Japan, e-cigarette devices and cartridges are sold at grocery stores without any restrictions [16]. E-cigarette use is also advertised as “e-cigarettes for smokers who want to quit smoking” and “quit cigarettes with e-cigarettes”. Almost all e-cigarette products are not associated with product descriptions and after services, nor are they associated with medical coaching systems, as is the case in the UK. Finding associations between e-cigarette use and quitting is an important concern in Japan. Therefore, our present research is a first step in building an evidence base that leads to policy changes with regard to e-cigarette use and advertising.

This study has several limitations. First, the selection of quitting method may depend on the balance between the participants’ motivation to stop smoking and their degree of dependence on cigarettes. Though e-cigarette use was negatively associated with smoking cessation, we did not assess whether e-cigarette use caused failure to quit smoking or whether those who had difficulty quitting were more likely to use e-cigarettes for smoking cessation. In addition, prescription medication combined with behavioral counseling was associated with increased cessation, whereas over-the-counter NRT had a lower association with smoking cessation [24]. We did not measure e-cigarette use intensity/duration and type. Therefore, we can interpret the observed negative association to mean that smoking status causes e-cigarette use, but not that e-cigarette use causes smoking cessation failure. Thus, it is unclear whether the association between smoking cessation therapy with varenicline and quitting is due to the efficacy of the drug, the comprehensive coaching process in place, or both. Second, because the sample size was relatively small, confidence intervals for ORs for some items such as quitting method remained wide. Besides, although the age–gender distribution was not biased by much, we did not weight the data in their analysis, so our findings may be sample-specific and may not be generalizable to the Japanese population. Third, all data were self-reported without validation. Although the reliability of self-reported smoking is generally considered high [3,25,26], reliability has not been confirmed for e-cigarettes or other heat-not-burn tobacco products. In addition, some participants may have reported inconsistent answers for current age, age of smoking initiation, and age of cessation, which would be a limiting factor for the overall accuracy of this investigation. Finally, we did not fully analyze the background and/or historical factors that might have influenced the tobacco addiction of our participants. In particular, it is unknown at which point in the process of their cessation that participants first tried the use of e-cigarettes and other cessation methods. The cross-sectional design of this study presents the problem of recall bias for retrospective components [27,28]. Participants may have found it difficult to remember or accurately retrieve actions made years in the past. Future research and follow-up studies are necessary to examine the prospective association between e-cigarette use and smoking cessation.

## 5. Conclusions

E-cigarette use was negatively associated with smoking cessation after adjustments for gender, age, health-related factors, and other cessation methods. Conversely, medical cessation therapy (i.e., varenicline) covered by the National Health Insurance program in Japan was significantly associated with smoking cessation in the same model. Since there is no present scientific consensus that e-cigarettes are beneficial for smoking cessation, any recommendation or promotion of e-cigarettes as a smoking cessation aid should be made with caution. Without strong evidence that e-cigarettes are efficacious in helping people to quit smoking, recommending them in the current manner is premature.

## Figures and Tables

**Table 1:** Characteristics of 798 persons who smoked within the previous 5 years according to present smoking status for combustible tobacco.

Characteristic	Total		Current Smoker		Former Smoker		*p*
Overall	No.	%	No.	%	No.	%	
	798	100.0	545	100.0	253	100.0	
Quitting methods used to date							
E-cigarette use	159	19.9	120	22.0	39	15.4	0.030
Over-the-counter NRT	217	27.2	151	27.7	66	26.1	0.632
Smoking cessation therapy for nicotine dependence	96	12.0	63	11.6	33	13.0	0.549
Varenicline	68	8.5	43	7.9	25	9.9	0.348
Others ^1^	56	7.0	39	7.2	17	6.7	0.822
Unassisted ^2^	666	83.5	446	81.8	220	87.0	0.070
Gender							0.451
Male	532	66.7	368	67.5	164	64.8	
Female	266	33.3	177	32.5	89	35.2	
Age group (years)							0.008
20–29	99	12.4	62	11.4	37	14.6	
30–39	177	22.2	113	20.7	64	25.3	
40–49	190	23.8	138	25.3	52	20.6	
50–59	181	22.7	139	25.5	42	16.6	
60–69	151	18.9	93	17.1	58	22.9	
Age started smoking cigarettes							0.237
<20 years	224	28.1	146	26.8	78	30.8	
≥20 years	574	71.9	399	73.2	175	69.2	
Marriage							0.003
Married	516	64.7	333	61.1	183	72.3	
Single	198	24.8	144	26.4	54	21.3	
Widowed/divorced	84	10.5	68	12.5	16	6.3	
Medical history							
Cancer and/or CVD	53	6.6	39	7.2	14	5.5	0.392
Hypertension	158	19.8	113	20.7	45	17.8	0.331
Diabetes Mellitus	61	7.6	45	8.3	16	6.3	0.339

E-cigarettes: electronic cigarettes; NRT: nicotine replacement therapy; CVD: cardiovascular disease; **^1^** Other smoking cessation therapies for nicotine dependence include cases with and without medical prescriptions containing nicotine, e.g., nicotine patches. **^2^** Unassisted refers to participants who tried to quit on their own (incl. utilization of books).

**Table 2:** Adjusted odds ratios (95% CIs) for smoking cessation within 5 years, according to quitting methods and socio-demographic factors, among 253 successful quitters and 545 unsuccessful quitters.

Variable	Model 1		Model 2	
	aOR	95% CI	aOR	95% CI
Quitting methods conducted to date ^1^				
E-cigarette use	**0.629**	**0.415–0.954**	**0.632**	**0.414–0.964**
Over the counter NRT	0.968	0.666–1.405	0.952	0.653–1.387
Smoking cessation therapy for nicotine dependence	-	-	-	-
Varenicline	1.747	0.958–3.188	**1.855**	**1.018–3.492**
Others	0.821	0.416–1.621	0.808	0.405–1.614
Unassisted	1.349	0.846–2.152	1.359	0.846–2.183
Gender				
Male (ref.)	1.000		1.000	
Female	1.100	0.795–1.523	1.160	0.828–1.627
Age group (years)				
20–29	1.687	0.996–2.855	**1.821**	**1.046–3.172**
30–39	1.502	0.961–2.346	1.554	0.981–2.460
40–49 (ref.)	1.000		1.000	
50–59	0.782	0.487–1.257	0.776	0.478–1.259
60–69	**1.737**	**1.091–2.763**	**1.793**	**1.085–2.963**
Age started smoking cigarettes				
<20 years (ref.)			1.000	
≥20 years			0.826	0.585–1.125
Marriage				
Married (ref.)			1.000	
Single			**0.604**	**0.404–0.901**
Widowed/divorced			**0.430**	**0.237–0.780**
Medical history				
Cancer and/or CVD			0.749	0.380–1.476
Hypertension			0.899	0.582–1.388
Diabetes Mellitus			0.713	0.375–1.358

**^1^** adjusted odds ratios (aORs) were analyzed compared with those who had never conducted each quitting method. Bolded values indicates statistical significance.

## Supplementary Materials

The following are available online at www.mdpi.com/1660-4601/14/2/202/s1, Table S1: Adjusted odds ratios (95% CIs) for smoking cessation within 4 and 3 years, according to quitting methods and socio-demographic factors.
