# Supplementary material for: Electronic Cigarette Use and Smoking Abstinence in Japan: A Cross-Sectional Study of Quitting Methods
Source: Int J Environ Res Public Health. 2017 Feb 17;14(2):202. doi: 10.3390/ijerph14020202 (PMC5334756; doi:10.3390/ijerph14020202)
Supplement: Supplementary file 1 [file ijerph-14-00202-s001.pdf]

Supplementary Materials: Electronic Cigarette Use and Smoking Abstinence in Japan: A Cross-Sectional Study of Quitting Methods

Tomoyasu Hirano, Takahiro Tabuchi, Rika Nakahara, Naoki Kunugita and Yumiko Mochizuki-Kobayashi

1. Supplementary Methods

We conducted a sensitivity analysis on the span width of most recent smoking history. Of the 798 persons who smoked within the previous five years and had considered quitting at least once, the analyses consisted of 756 and 709 persons who smoked within the previous 4 and 3 years, respectively. They smoked within the previous 4 and 3 years and had considered quitting at least once within the previous 4 or 3 years, respectively.

2. Supplementary Results

Results of logistic regression models for smoking cessation among smokers within the previous 4 and 3 years are shown in Table S1. As in Table 2, showing smokers within the previous 5 years, e-cigarettes use was negatively associated with smoking cessation after adjustment for potential confounding factors (aOR = 0.598, 95% CI = 0.377–0.949, within 4 years; aOR = 0.638, 95% CI = 0.385–1.056, within 3 years). Statistical significance was diminished in an analysis using recent 3-year smokers, although this result might attributable to the small sample size of the smokers ($n = 709$).

Table S1. Adjusted odds ratios (95% CIs) for smoking cessation within the previous 4 and 3 years, according to quitting methods and socio-demographic factors.

Variable	4 Years (*n* = 756)		3 Years (*n* = 709)	
	aOR	95% CI	aOR	95% CI
**Quitting methods conducted to date** [1]				
E-cigarette use	0.598	0.377–0.949	0.638	0.385–1.056
Over-the-counter NRT	0.867	0.568–1.322	0.890	0.557–1.422
Smoking cessation therapy for nicotine dependence	-	-	-	-
Varenicline	1.929	0.971–3.832	1.798	0.847–3.818
Others	0.823	0.380–1.783	0.916	0.395–2.125
Unassisted	1.471	0.859–2.520	1.575	0.867–2.861
**Gender**				
Male (ref.)	1.000		1.000	
Female	1.143	0.792–1.648	1.049	0.700–1.573
**Age group (years)**				
20–29	2.571	1.453–4.550	2.592	1.387–4.844
30–39	1.761	1.073–2.888	1.926	1.123–3.304
40–49 (ref.)	1.000		1.000	
50–59	0.659	0.381–1.138	0.658	0.358–1.211
60–69	1.547	0.891–2.688	1.356	0.729–2.519
**Age smoking cigarettes started**				
<20 years (ref.)	1.00		1.000	
≥20 years	0.857	0.590–1.245	0.788	0.527–1.178

Table S1. *Cont.*

Variable	4 Years (*n* = 756)		3 Years (*n* = 709)	
	**aOR**	**95% CI**	**aOR**	**95% CI**
**Marriage**				
Married (ref.)	1.000		1.000	
Single	0.539	0.352–0.828	0.538	0.337–0.860
Widowed/divorced	0.325	0.159–0.665	0.348	0.159–0.762
**History of disorder**				
Cancer and/or CVD	0.651	0.300–1.409	0.812	0.359–1.835
Hypertension	1.097	0.689–1.749	1.015	0.596–1.728
Diabetes Mellitus	0.770	0.392–1.513	0.806	0.381–1.705

E-cigarettes: electronic cigarettes; NRT: nicotine replacement therapy; CVD: cardiovascular disease; [1] aORs were analyzed compared with those who never conducted each method to quit, as references.

© 2017 by the authors; licensee MDPI, Basel, Switzerland. This article is an open access article distributed under the terms and conditions of the Creative Commons by Attribution (CC-BY) license (http://creativecommons.org/licenses/by/4.0/).
